# Dysfunctional breathing phenotype in adults with asthma - incidence and risk factors

**DOI:** 10.1186/2045-7022-2-18

**Published:** 2012-09-19

**Authors:** Ioana Agache, Cristina Ciobanu, Gabriela Paul, Liliana Rogozea

**Affiliations:** 1 Department of Allergy and Clinical Immunology, Transylvania University, Faculty of Medicine, 56 Nicolae Balcescu, Brasov, Romania; 2 Theramed Medical Center, Department of Allergy and Clinical Immunology, 16 Spatarul Luca Arbore, Brasov, Romania

**Keywords:** Dysfunctional breathing, Asthma, Co-morbidities, Phenotype

## Abstract

**Background:**

Abnormal breathing patterns may cause characteristic symptoms and impair quality of life. In a cross-sectional survey 29% of adults treated for asthma in primary care had symptoms suggestive of dysfunctional breathing (DB), more likely to be female and younger, with no differences for severity of asthma. No clear risk factors were demonstrated for DB in asthma, nor the impact of asthma medication was evaluated. The objective of this study was to describe the DB phenotype in adults with asthma treated in a specialised asthma centre.

**Methods:**

Adult patients aged 17–65 with diagnosed asthma were screened for DB using the Nijmegen questionnaire (positive predictive score >23) and confirmed by progressive exercise testing. The following were evaluated as independent risk factors for DB in the multiple regression analysis: female sex; atopy, obesity, active smoker, moderate/severe rhinitis, psychopathology, GERD, arterial hypertension; severe asthma, asthma duration > 5 years, lack of asthma control, fixed airway obstruction, fast lung function decline, frequent exacerbator and brittle asthma phenotypes; lack of ICS, use of LABA or LTRA.

**Results:**

91 adults with asthma, mean age 35.04 ±1.19 years, 47(51.65%) females were evaluated. 27 (29.67%) subjects had a positive screening score on Nijmegen questionnaire and 16(17.58%) were confirmed by progressive exercise testing as having DB. Independent risk factors for DB were psychopathology (p = 0.000002), frequent exacerbator asthma phenotype (p = 0.01) and uncontrolled asthma (p < 0.000001).

**Conclusion:**

Dysfunctional breathing is not infrequent in asthma patients and should be evaluated in asthma patients presenting with psychopathology, frequent severe asthma exacerbations or uncontrolled asthma. Asthma medication (ICS, LABA or LTRA) had no significant relation with dysfunctional breathing.

## Introduction

Dysfunctional breathing (DB) is defined as chronic or recurrent changes in breathing pattern that cannot be attributed to a specific medical diagnosis, causing respiratory and non-respiratory complaints such as anxiety, light headedness and fatigue. Symptoms of DB include dyspnea with normal lung function, chest tightness, chest pain, deep sighing, exercise-induced breathlessness, frequent yawning and hyperventilation [1-6]. There is no gold standard for the diagnosis of DB beyond the clinical description. The Nijmegen Questionnaire can be used to discriminate dysfunctional breathers from normal individuals in adults [7]. For moderate to severe asthma a positive Nijmegen score might overestimate the presence of DB and confirmation via progressive exercise testing was suggested [8].

DB associates significant morbidity in the inflicted subjects. Compared to patients with well controlled asthma DB subjects had significantly lower health-related quality of life (Short Form 36), a higher prevalence of anxiety (56% vs 24%) and a lower sense of coherence [9]. Asthmatic subjects with co-existing DB had lower quality of life scores compared to those without DB [8].

Several breathing therapies are used to manage DB. The most popular ones are the Buteyko breathing method, pursed lip breathing and traditional Hatha yoga. All breathing therapies focus on strengthening the diaphragmatic breathing during breath work, some also address lifestyle and have strict criteria for success/progress (Hatha Yoga and Buteyko method). Most breathing therapies need an instructor for a successful learning. Patients are instructed to practice exercises at home and when symptomatic [10].

Although DB diagnosis is done by excluding a specific cause of dyspnea, it can co-exist with asthma, chronic obstructive pulmonary disease (COPD) or sensory hyper-reactivity. No technique has yet been validated to identify DB in the presence of other respiratory disease such as asthma, although a recent report suggests that the combination of Nijmegen questionnaire as a screening tool followed by progressive exercise testing might increase the diagnostic specificity for the identification of DB in asthma patients [8].

In a primary care setting Nijmengen score was used to evaluate the incidence of symptoms suggestive of DB in adults with or without asthma. The incidence of DB was significantly higher in asthmatics (29%) compared to subjects without asthma (8%) [11]. The study had a cross-sectional design and DB was diagnosed solely based on positive screening score on Nijmegen questionnaire. Conversely, 80% of subjects diagnosed as hyperventilation syndrome had asthma [12].

Unlike for COPD [13,14], there is no clear cut relation between disease physiopathology and the appearance of DB in asthma. The increased incidence of DB in asthma may to attributed to several factors, from misdiagnosis of DB as asthma to the potential contribution of over-breathing to the airway inflammation [15] and hyperreactivity [15,16]. DB may contribute significantly to symptoms in asthma and thus result in over-prescription of drug treatment.

No clear risk factors were identified for DB in asthma. Asthmatic DB subjects were more likely to be female and younger, with no differences for severity of asthma [11,17]. The impact of asthma medication on DB incidence was not evaluated.

The aim of this study was to describe the incidence and risk factors for the dysfunctional breathing phenotype in adults with asthma evaluated in a specialized asthma centre. Our hypothesis was that the incidence of DB will vary according to the diagnostic method used and that several phenotypic traits and asthma co-morbidities can define asthma subjects prone to dysfunctional breathing.

## Material and methods

Study protocol was approved by the local ethics committee and all subjects provided written informed consent before entering the trial. To avoid selection bias only 25% of subjects were randomly picked up from our own database, while 75% were referred by five other asthma clinics.

To be selected into the trial the subjects had to comply with the following *inclusion criteria*: adults with asthma, defined by history and an FEV1 increase after a bronchodilator > 12% and 200 ml at inclusion; provision of informed consent; willingness to comply with study procedures; available detailed data on asthma history (including control, severity and exacerbations), asthma treatment and serial lung function measurement in the previous 12 months.

At inclusion the patients for evaluated for demographics and asthma background (history, treatment, previous lung function measurements). Lung function with bronchodilator testing (Micromedical MK8) was performed to confirm the diagnosis of asthma. DB was identified by a positive screening score > 23 on Nijmegen questionnaire and confirmed via progressive exercise testing.

A symptom-limited exercise test using one cycle ergometer. was performed using a stepwise increase in work load of 16 W·min^-1^, starting with unloaded cycling [18]. All tests were continued as symptom-limited (dyspnea), in the absence of chest pain or ECG abnormalities. Prior to the test, patients were encouraged to reach maximal exhaustion, while during exercise no further motivational interventions were utilised. The procedure was continuously monitored by a physician. All tests were performed in room air according to current guidelines for exercise testing, with continuous monitoring of ECG, blood pressure and oxygen saturation. The presence of an abnormal breathing pattern such as an increase in deep sigh rate in response to exercise or unsteadiness and irregularity of breathing with no evidence of bronchoconstriction on spirometry and good exercise tolerance was considered diagnostic for DB. The abnormal breathing pattern was assessed by the same physician for all the subjects evaluated, in comparison with the baseline breathing pattern.

The following *risk factors for DB* were evaluated at inclusion:

A. demographic features: age, female sex

B. Asthma co-morbidities: obesity; atopy; active smoker status; moderate/severe rhinitis; psychopathology; gastro-esophageal reflux (GERD); high blood pressure;

C. Asthma phenotypic traits: severe asthma; lack of asthma control; asthma duration > 5 years; fixed airway obstruction; fast lung function decline; frequent exacerbator phenotype; brittle asthma type I and II;

D. Asthma medication: lack of ICS use in the last 6 months; use of long acting beta 2 agonists (LABA) as add-on to ICS in the last 6 months; use or leukotriene receptor antagonists (LTRA) in monotherapy or as add-on to ICS in the last 6 months.

Obesity was defined by a BMI at or above the 95th percentile. Atopy was diagnosed if at least one skin prick test (SPT) was positive from the common aeroallergens tested (house dust mites, cat, dog, molds, cockroaches, grass/trees/weeds pollen). SPT was considered positive if for a wheal diameter of allergen/wheal diameter of histamine > 1 and a mean wheal size ≥ 3 mm. Active smoker status was defined as daily smoking. Moderate/severe rhinitis was diagnosed using the ARIA criteria for severity [19], in association with an ENT examination excluding other nasal pathologies. A careful examination by an adult psychologist was performed. Anxiety or depression (Hospital Anxiety and Depression Scale score > 11) were considered as psychopathologic risk factors for DB. According to the National Institute of Mental Health anxiety disorders were separated into generalized anxiety disorder, obsessive-compulsive disorder, panic disorder, post-traumatic stress disorder and phobias, including the specific phobia of blood, injections, and injuries. GERD was diagnosed by gastro-esophageal endoscopy. High blood pressure was diagnosed by ambulatory blood pressure monitoring as values above 135/85 mmHg awake and above 120/75 mmHg during sleep [20]. Severe asthma was defined according to the recommendations of World Health Organization Consultation on Severe Asthma [21]. Lack of asthma control (partial or non-controlled asthma) was defined using the GINA criteria [22]. Fixed airway obstruction was considered for an FEV1 < 80% predicted despite maximal therapy in the 12 months at all serial lung function measurements performed. Fast lung function decline was defined as an FEV1 decrease >100 ml/year in previous 12 months. The frequent exacerbator phenotype was considered in the presence of at least 3 exacerbations requiring systemic steroids and/or ER visit and/or hospitalization for asthma in the previous 12 months. Brittle asthma type I was diagnosed based on more than 40% diurnal variability in PEF rate on most days over previous 6 months despite maximal treatment, while type II brittle asthma was considered for patients with acute severe attacks on a background of apparently good asthma control [23].

### Statistical analysis

Results were analyzed with STATISTICA 7 (StatSoft. Inc, Tulsa, USA). The differences between asthma patients groups with and without DB were tested using the Chi-test and *t*-test for independent samples. Independent risk factors for DB were analyzed in the multiple regression analysis. To avoid falsely positive conclusions, the significance level at each test was set to 0.05.

## Results

200 adults asthma patients were screened and 91 asthmatics fulfilling all the inclusion criteria were enrolled. Mean age of enrolled asthmatics was 35.04 ±1.19 years and 47(51.65%) were females.

27 (29.67%) asthma patients had a positive screening score (>23) after completing the Nijmegen questionnaire and 16(17.58%) were confirmed by progressive exercise testing as having DB.

The incidence of risk factors evaluated for DB is presented in Table 1. In the group of asthma patients with DB there was increased incidence of female sex and of asthma co-morbidities such as moderate/severe rhinitis, psychopathology and GERD. Anxiety disorders were diagnosed in 7 of the subjects from the DB group, followed by depression in 4 cases, while in the non DB group both cases were diagnosed with depression. Out of the 7 anxiety disorders identified 5 were panic disorder and 2 were specific phobia of blood, injections, and injuries.

**Table 1:** Incidence of risk factors for dysfunctional breathing in study group

***Risk factor***	***Subjects with DB (n = 16)***	***Subjects without DB (n = 75)***	***p value***
*Age (years; mean ± SD)*	*37.88 ± 1.39*	*34.44 ± 1.39*	*0,2880*
*Female sex*	13(82.25%)*	34(45.33%)	0,0091
*Obesity*	6(37.5%)	37(49.33%)	0,3894
*Atopy*	9(56.25%)	72(96%)*	<0,00001
*Active smoker*	5(31.25%)	12(16%)	0,1554
*Moderate/severe rhinitis*	13((82.25%)*	41(54.67%)	0,0494
*Psychopathology*	12(75%)*	2(2.67%)	<0,00001
*GERD*	8(50%)*	1(1.33%)	<0,00001
*High blood pressure*	2(12.5%)	6(8%)	0,5639
*Severe asthma*	9(56.25%)*	15(20%)	0,0028
*Lack of asthma control*	13((82.25%)*	4(5.33%)	<0,00001
*Asthma duration > 5 years*	9(56.25%)	25(33.33%)	0,0854
*Fixed airway obstruction*	1(6.25%)	11(14.67%)	0,3663
*Fast FEV1 decline*	4(25%)*	4(5.33%)	0,0117
*Frequent exacerbator*	15(93.75%)*	8(10.67%)	<0,00001
*Brittle asthma*	6(37.5%)*	1(1.33%)	<0,00001
*Lack of ICS*	4(25%)	44(58.67%)*	0,0143
*Use of LABA as add-on*	7(43.75%)	16(21.33%)	0,0611
*Use of LTRA*	7(43.75%)	55(73.33%)*	0,0211

* p value statistically significant.

In the DB group there was also significant increased incidence of severe asthma, lack of asthma control and of asthma phenotypes fast FEV1 decline, frequent exacerbator and brittle asthma. Conversely, atopy was significantly higher in the asthma patients group without DB, together with lack of use of ICS and use of LTRA, either as monotherapy or as add-on to inhaled corticosteroids.

In the multiple regressions analysis however only psychopathology, lack of asthma control and the frequent exacerbator phenotype were independent risk factors for DB (Table 2).

**Table 2:** Risk factors for dysfunctional breathing (multiple regression analysis)

***Risk factor***	***Beta***	**St.Err of beta**	**B**	***S.Err of B***	***p value***
*Female sex*	0.044035	0.053897	0.032830	0.040182	0.416651
*Obesity*	−0.027895	0.054722	−0.020812	0.040828	0.611803
*Atopy*	0.039556	0.056458	0.046908	0.066951	0.485822
*Active smoker*	−0.044177	0.052323	−0.042061	0.049817	0.401331
*Moderate/severe rhinitis*	0.086165	0.054499	0.065263	0.041278	0.118311
*Psychopathology*	0.395755	0.076665	0.406942	0.078832	0.000002*
*GERD*	0.087420	0.080967	0.103668	0.096015	0.283928
*High blood pressure*	−0.011727	0.054169	−0.016319	0.075377	0.829222
*Severe asthma*	−0.057307	0.074725	−0.048295	0.062974	0.445681
*Lack of asthma control*	−0.437868	0.072179	−0.407959	0.067249	<0.0000001*
*Asthma duration > 5 years*	−0.038431	0.058607	−0.029108	0.044390	0.514114
*Fixed airway obstruction*	−0.073994	0.053388	−0.081121	0.058531	0.170100
*Fast FEV1 decline*	0.039443	0.057331	0.051653	0.075078	0.493702
*Frequent exacerbator*	0.177586	0.070043	0.151735	0.059847	0.013436*
*Brittle asthma*	−0.024448	0.063002	−0.036526	0.094127	0.699140
*Lack of ICS*	0.025523	0.071903	0.019043	0.053646	0.723664
*Use of LABA as add-on*	0.031491	0.094519	0.025350	0.076089	0.739988
*Use of LTRA*	0.068785	0.051638	0.056558	0.042459	0.187104

* p value statistically significant.

## Discussion

### Incidence of dysfunctional breathing

The incidence of a positive Nijmegen screening score for DB in our study was 29.67%, matching perfectly with the previous UK study [11,17] where DB was diagnosed solely based on Nijmegen questionnaire. However, only 16 cases (59.3% of the patients with a positive screening score) showed inappropriate hyperventilation at the progressive exercise testing and thus were confirmed with DB. The result is similar to that reported by Stanton et al. [8], where only 58.8% of patients with a positive Nijmegen screening score were confirmed as having DB.

The results are confirming our hypothesis that different diagnostic methods report different incidences for DB. Correct diagnosis of DB in asthma patients is of paramount importance. Some of the asthma symptoms such as breathlessness, chest pain, chest constriction and accelerated breathing overlap with DB symptoms as depicted by Nijmegen questionnaire. Unlike other diseases causing breathlessness the diagnosis of DB is not based on independent criteria, thus the Nijmegen questionnaire is different from other disease specific health status measures [24]. In the original validation of the Nijmegen questionnaire there was some crossover with a healthy reference group [7] and no validation was performed with people who had other causes of dyspnea. Progressive exercise testing may depict inappropriate ventilation, but hypocapnia may not be always detected [25] and a standard set of diagnostic parameters is lacking. In our study we diagnosed inappropriate ventilation case by case in comparison with the baseline breathing pattern and in relation to objective parameters such as work load and lung function. Other proposed standardized methods for diagnosing DB are the Respiratory Induction Plethysmography (RIP) and a technique evaluating and quantifying the breathing pattern, called the Manual Assessment of Respiratory Motion (MARM). Both RIP and MARM methods were able to differentiate between abdominal and thoracic breathing patterns, but only MARM was able to differentiate between breathing changes occurring as result of slumped versus erect sitting posture [26]. However, none of these techniques are validated for asthma patients.

### Dysfunctional breathing and asthma co-morbidities

The present study is the first to evaluate the relation between DB and other asthma co-morbidities. Except psychopathology, other asthma co-morbidities evaluated (obesity, moderate/severe rhinitis, GERD, atopy, high blood pressure) did not increase the risk for DB in the multiple regression analysis. However, there was increased incidence of moderate/severe rhinitis and GERD in the DB group (Table 1), suggesting a careful examination for DB in patients with asthma associated with these two co-morbidities. The increased incidence of moderate/severe rhinitis in asthmatic DB patients is not surprising considering the consequences of oral breathing and the link between rhinitis and asthma, which might involve common abnormal neural pathways. In a study evaluating patients complaining of ongoing nasal congestion, despite an apparently adequate surgical result and appropriate medical management, there was significant increased incidence of an elevated respiratory rate, with an upper thoracic breathing pattern and an elevated Nijmegen score [27]. The nasal congestion could be the consequence of the abnormal breathing pattern with low PaCO2 levels increasing nasal resistance. Another possible explanation is reduced alae nasae muscle activity secondary to the reduced activity of serotonin-containing raphe neurons [27]. The relation between DB and GERD is an intriguing observation which deserves further evaluation, since it has been shown in asthmatic subjects that perfusion of acid into the distal esophagus increases the bronchoconstriction produced by isocapnic hyperventilation and by methacholine [28]. The increased incidence of atopy in the non-DB asthma patients group might suggest that DB appears more frequently in non-atopic asthma. The lack of association between DB and obesity is not surprising since hypoventilation is significantly more frequently encountered in obese patients [29] and an improvement in compensatory hyperventilation during exercise is closely related to loss in overall fat mass [30].

The major asthma co-morbidity (Table 2) increasing the risk for DB in asthma patients are the anxiety disorders, especially panic disorders and specific phobia of blood, injections, and injuries. Both panic disorders and specific phobias were related to asthma [31,32], and hyperventilation may provide an interesting link with asthma, based on sustained levels of hypocapnia [33,34]. In patients with panic disorders raising CO2 levels by therapeutic capnometry proved superior to cognitive-behavior therapy [33]. The method is not yet validated for asthma, since it was tested only in a small trial [35]. However, a larger clinical trial sponsored by the National Heart, Lung and Blood Institute will evaluate the benefits of therapeutic capnometry for asthma patients, with a particular focus on changes in PaCO2 and inflammation [33].

### Dysfunctional breathing and asthma phenotypic traits

We also examined the relation between phenotypic traits of asthma and DB. Lack of asthma control and the frequent exacerbator phenotype were independent predictors for DB in the multiple regression analysis.

As any other asthma co-morbidity DB is closely related to asthma control. However, specific studies aiming at DB treatment in asthma patients focused solely on the quality of life status, which is only an indirect indicator for asthma control [36]. In an interventional study breathing retraining did not improve the objective measures of respiratory function except for relaxed breathing rate [37]. In the presence of DB it is imperative to combine multiple measures for assessing asthma control, especially lung function measurement. In a case report of two children with asthma and DB the authors reported on the low Asthma Control Test score in the absence of asthma worsening [38]. As indicated by GINA guidelines [22] lack of control in our study was defined by a composite measure of daytime and nighttime symptoms, limitation of activities, need for reliever medication and lung function testing.

The association of the frequent exacerbator phenotype with DB in asthma is an interesting observation of this study, since we described this phenotype as an asthmatic with at least 3 severe exacerbations requiring systemic steroids and/or ER visit and/or hospitalization for asthma in the previous 12 months. The presence of DB should be carefully diagnosed in these patients in order to avoid over-treatment and to allow patient to benefit from non-pharmacological interventions.

In the DB group there was significant increased incidence of severe asthma and of brittle asthma. These two asthma phenotypes could also benefit from non-pharmacological treatment and possible prevention of asthma attacks if DB is correctly and promptly identified. Since fast FEV1 decline was encountered more frequently in the DB group, the condition might prove important for evaluating asthma future risk.

### Relation with asthma medication

Asthma medication (LABA or LTRA) associated no increased risk for DB in the multiple regression analysis. Lack of ICS in the past six months, as a measure of under treatment of steroid-responsive asthmatic inflammation, was not associated with an increased risk for DB. Since in the non-DB group there was an increased usage of LTRA as a first line controller (Table 1), exploring the leukotriene pathway in the pathogenesis of hyperventilation syndrome might prove of interest. As an alternative explanation there were significantly more milder asthmatics in the non-DB group compared to the DB group (Table 1).

### Limitations and open questions

This study is obviously hampered by the non-interventional design and by the small number of subjects included. However, it was carefully planned as a proof of concept study and the hypothesis of a special risk profile for dysfunctional breathing phenotype in an asthmatic patient was proved. Further validation of our findings in a prospective, interventional trial is warranted in order to clarify the role of DB in relation to asthma co-morbidities and phenotypic traits.

The lack of a standard set of diagnostic parameters to define inappropriate ventilation during progressive exercise testing is also acknowledged. However, as previously explained, we tried to eliminate as much as possible the subjective character of the evaluation by defining inappropriate ventilation case by case in comparison with the baseline breathing pattern and in relation to objective parameters such as work load and lung function. There is a clear and urgent need to validate tests such as progressive exercise testing, RIP or MARM for diagnosis of DB in asthma patients.

### Summary

This study shows that dysfunctional breathing is not infrequent in adults with asthma and should be evaluated in asthma patients presenting with, frequent severe asthma exacerbations and uncontrolled asthma.

The occurrence of dysfunctional breathing in asthma offers an explanation for asthma symptoms from a different perspective. Correct and prompt identification of abnormal breathing patterns in asthma offers the opportunity for treatment beyond pharmacology and for reducing unnecessary medication to control asthma symptoms. The main issue to be solved remains the validation of a gold-standard method for diagnosing dysfunctional breathing in asthma patients by using objective measurements.

## Abbreviations

ACT: Asthma Control Test; ARIA: Allergic Rhinitis and its Impact on Asthma; BM: Body Mass Index; COPD: Chronic Obstructive lung Disease; DB: Dysfunctional Breathing; ECG: Electrocardiogram; ENT: Ear/Nose/Throat; ER: Emergency Room; FEV1: Forced Expiratory Volume in the first second; GERD: Gastro-oesophageal reflux disease; GINA: Global Initiative for Asthma; ICS: Inhaled Corticosteroids; LABA: Long-acting Beta2 Agonists; LTRA: Leukotriene Receptor Antagonists; MARM: Manual Assessment of Respiratory Motion; SPT: Skin Prick Test; PaCO2: Partial Pressure of Carbon Dioxide in the arterial blood; RIP: Respiratory Induction Plethysmography.

## Competing interests

The authors declare no competing interests relevant to this paper.

## Authors’ contribution

IA, LR – study design and coordination. CC, GP, LR – patient evaluation and laboratory work. IA, CC, LR – data analysis and interpretation of results. IA – preparation and critical revision of the manuscript. All authors read and approved the final manuscript.
